# Clinician Opinions Regarding the Usefulness of the BOSA for ASD Assessment in a Service for Children Aged Under 12 Years

**DOI:** 10.1007/s10803-023-06207-z

**Published:** 2024-04-02

**Authors:** Sophie Shapter, Amy Carroll, Kate Roberts

**Affiliations:** 1 Faculty of Medicine and Health Sciences, University of East Anglia, Norwich Research Park, Norwich, NR4 7TJ UK; 2 Oak Tree Centre, Cambridgeshire Community Services NHS Trust, Huntingdon, PE29 7HN UK

**Keywords:** Autism spectrum disorder, COVID-19 pandemic, BOSA, ADOS-2, Telemedicine

## Abstract

The COVID-19 pandemic and the subsequent social distancing policies forced healthcare teams to drastically alter the way they deliver services. This was particularly challenging for clinicians involved in diagnosing autism spectrum disorder (ASD), as assessment tools and methods required face-to-face social interactions between clinicians and children. To address this, the Brief Observation of Symptoms of Autism (BOSA) was developed to ensure that people suspected of ASD can receive diagnostic assessments during the pandemic. This project aimed to explore clinicians’ opinions on the BOSA, particularly regarding the usefulness of the assessment for clinicians to clarify diagnostic outcomes of ASD assessments. Both quantitative and qualitative data was gathered within an NHS community paediatric team. This included a questionnaire for clinicians to complete, and data from the BOSA assessments done in the service. Thematic analysis and descriptive statistics revealed that many clinicians felt that the BOSA can be beneficial in certain cases, such as selective mutism, and found the BOSA particularly helpful for observing parent–child interactions. These findings highlighted important information that the Autism Diagnostic Observation Schedule Second Edition (ADOS-2) does not give opportunities to observe. Clinicians reported that at times, the BOSA materials, brevity and parental administration created barriers to gathering information for diagnostic decisions. As may be expected, clinicians showed a clear preference for the more familiar and validated ADOS-2. However, the study highlights perceived limitations of the ADOS-2 and strengths of the BOSA, with recommendations made for future practice and research.

## Introduction

The coronavirus (COVID-19) pandemic has imposed major changes to the delivery of healthcare worldwide. In many settings, healthcare has been predominantly transitioned to telehealth (via phone or video calls), creating challenges but also unique benefits. This transition was particularly challenging for services assessing children for neurodevelopmental disorders such as autism spectrum disorder (ASD; Zwaigenbaum et al., 2021).

To reliably diagnose ASD, one of the key areas that needs to be assessed is social communication and interaction (American Psychiatric Association, 2013). Information is often gathered through an account of an individual’s developmental history, school reports and behavioral observations. Prior to COVID-19, clinicians would often use the Autism Diagnostic Observation Schedule Second Edition (ADOS-2; Lord et al., 2012) as an observational tool to gather information, particularly regarding social communication and interaction skills.

The ADOS-2 is a structured play-based assessment designed for face-to-face, distraction-free settings using standardised objects and toys. Clinicians must be trained in ADOS-2 administration and scoring to conduct an ADOS-2 assessment. Due to the necessity for a reliable ADOS-2 to be in-person and for items to be touched by both client and clinician, the ADOS-2 is not COVID-19 secure. Additionally, the United Kingdom (UK) government COVID-19 safety regulations and National Health Service (NHS) policies for using personal protective equipment (PPE), (especially wearing face masks), are not only far from the ADOS-2 standardised procedures, they create barriers to establishing the interpersonal context required to assess certain ASD symptoms (Berger et al., 2022).

In response to COVID-19, the developers of the ADOS-2 created the Brief Observation of Symptoms of Autism (BOSA; Lord et al., 2020) to ensure patients suspected of ASD could receive safe and comprehensive diagnostic assessments. The BOSA is based on standardised activities selected and adapted from two validated ASD assessment tools: the ADOS-2 (Lord et al., 2012) and the Brief Observation of Social Communication Change (BOSCC; Grzadzinski, 2018; Grzadzinski et al., 2016).

The social interaction activities used within the BOSA provide opportunities to create social situations in which clinicians can observe symptoms of ASD within a 12-to-14-min observation (Lord et al., 2020). There are four separate modules of the BOSA that can be administrated depending on an individual’s age and language level. The BOSA-MV (minimally verbal) is appropriate for an individual of any age who is nonverbal or uses only single words or phrases. The BOSA-PSYF (phrase speech and young fluent) can be chosen for verbally fluent children aged six to eight. The BOSA-F1 (fluent speech F1) is designed for verbally fluent children aged between six to eight and 10 years old. The BOSA-F2 (fluent speech F2) is appropriate for verbally fluent individuals aged 11 and older, including adults. The latter two modules are very similar, except for the materials and questions being adapted to be age appropriate.

Unlike the ADOS-2, the BOSA is designed to be facilitated by any adult able to interact with a child without PPE (for example the child’s family member) to comply with COVID-19 restrictions. During the assessment, an instruction sheet is given to this adult to show them how to present and lead the child through the structured activities using the toys and objects provided. ADOS-2 trained clinicians will observe, and this can be done in a variety of ways, including through a one-way mirror in an observation room, via a video call or by being in the same room and maintaining the required social distancing and PPE. Once the BOSA is completed, the clinicians evaluate any ASD symptoms observed based on the BOSA scoring guidelines written by Lord et al., (2020).

ASD telehealth is a rapidly evolving field, and it is important to note that at the time of writing this report, the BOSA is in the initial stages of psychometric evaluation. Dow et al. (2021) have developed some algorithms to improve the psychometrics of the BOSA and other research may be investigating predictive effectiveness (Rynkiewicz et al., 2020).

The BOSA is not validated yet, and so the sensitivity and specificity of the assessment is unknown, and while there are recommended cut-off scores these are preliminary at present (Dow et al., 2021; Rynkiewicz et al., 2020). Lord et al., (2020) also highlight additional clinical considerations for clinicians. For example, due to the structured activities in the BOSA, certain symptoms may not be as readily apparent (e.g., restricted/repetitive behaviours and interests) as they are throughout the ADOS-2. Because of this and the brevity of the observation period there is a possible risk for “false negatives” (i.e., ruling out ASD when the person does have ASD; Lord et al., 2020).

However, clinicians can gather a lot of information from observing interactions between a parent and child, even if this is not formally scored in the context of the BOSA activities (Lord et al., 2020). An additional benefit of the BOSA is that it takes approximately 30 min to administer, which is significantly shorter when compared to the ADOS-2, which is often conducted over an hour-long appointment. This is a big advantage in already stretched healthcare systems where waiting lists for ASD assessments are exceeding target waiting times (NHS England, 2019; Jayanetti, 2022). Long waits for assessments are likely to cause negative diagnostic experiences, increased levels of stress and reduced levels of satisfaction in the diagnostic process (Crane et al., 2016; Howlin & Moore, 1997; Mori et al., 2009) as well as delaying parents and children accessing support (Mansell & Morris, 2004). Thus, if the BOSA is more time efficient and as useful for clinicians as the ADOS-2, it would be advantageous for services to consider taking forward.

Prior to the pandemic, telehealth ASD assessments had been developed and were being used, particularly for children in rural and low-socioeconomic communities who struggled to access ASD assessments (Nazneen et al., 2015; Smith et al., 2017). The Naturalistic Observation Diagnostic Assessment (NODA) is a store-and-forward telehealth approach to ASD diagnosis that relies on parents and care givers sharing video recordings of live events with clinicians for review and assessment. This approach enables families to record videos in their home during their day-to-day activities over several days, capturing a wider range of behaviours compared to a single clinic-based or live telehealth assessment (Smith et al., 2017). Research has shown diagnostic decisions made after completing a NODA are similar to those made after an in-person assessment (Smith et al., 2017).

Additionally, the Childhood Autism Rating Scale, Second Edition (CARS-2) was developed prior to the pandemic (Schopler et al., 2010). The CARS-2 is a standardised clinician observational tool for autism symptoms in children. Clinicians complete a 15-item rating scale after observing the child’s behaviour. This is scored up and can help to identify children with autism and determine symptom severity through quantifiable ratings based on the observation and a parent interview. As with the BOSA, this can be done face-to-face or via telehealth over a videoconferencing platform (Berger et al., 2022).

However, before COVID-19, virtual ASD assessment methods were not commonly used within the NHS. In addition to the BOSA, other telehealth ASD assessments were developed in response to the pandemic, such as the TELE-ASD-PEDS (Corona et al., 2020). The TELE-ASD-PEDS was created to allow clinicians to remotely observe interactions (roughly lasting 15–20 min) between caregivers and their children to help with diagnostic decision-making. The tool was designed for children under 36 months of age and involves a range of eight activities designed to provide clinicians opportunities to assess any potential symptoms and behaviours related to ASD. Although there are benefits to the TELE-ASD-PEDS tool (e.g., reduced travel time and costs, familiarity of environment and people), certain limitations were noted (Wagner et al., 2021). Technological barriers such as the difficulty of capturing certain behaviours on the camera (e.g. eye contact), unstable internet connections and lack of familiarity with the video conferencing software caused challenges for clinicians (Corona et al., 2021; Wagner et al., 2021). It is unclear as to whether the TELE-ASD-PEDS has been administrated in person whilst complying to COVID-19 regulations as the BOSA has, as this methodology could eliminate several technological limitations. Additionally, the age range for the TELE-ASD-PEDS is significantly smaller than that of the BOSA.

Another recently developed virtual ASD assessment tool The Adapted Virtual Autism Behavior Observation (A-VABO; Kryszak & Albright, 2020). The A-VABO invites caregivers to interact with their child by facilitating 15 activities following a script. Unlike the BOSA, the family are able to use their own toys and games, although some activities require specific items which for some families (particularly of lower socio-economic backgrounds) can become a barrier to administration (Berger et al., 2022). Furthermore, the reading level for the A-VABO script and instructions is deemed five grades higher than that of the BOSA which again can cause challenges for facilitating caregivers and clinicians.

This paper reports on an NHS community paediatric service in England who, like many services, started using the BOSA in response to COVID-19. The BOSA was chosen due to the similarity to the ADOS-2, for which all clinicians were already trained. This also made the BOSA cost-effective for the NHS (e.g., ADOS-2 toys and games were compatible, the training and materials were free to those with prior ADOS-2 training). As some COVID-19 restrictions were being lifted, the service wanted to evaluate how useful clinicians were finding the BOSA for diagnostic decision making and whether it should continue to be used.

This project will use quantitative and qualitative data gathered within an NHS community paediatrics team. The project aims to capture clinicians’ views of using the BOSA and explore if it is useful to clarify diagnostic decisions. The results of this project will help inform services about the continued use of the BOSA.

## Methods

### Design

This project used a mixed methods design comprising of two components to explore the usefulness of the BOSA for diagnostic decision making: (1) forced-choice questions related to clinicians’ opinions on the BOSA; and (2) a thematic analysis of open-ended questions related to clinicians’ opinions of the BOSA.

### Service Context

The study was completed within an NHS community paediatrics team in the East of England, commissioned to assess ASD in children under 12.

Children were referred to the service most commonly by their school’s special educational needs coordinator. Following this, teachers and parents/caregivers would complete the Social Communication Questionnaire (SCQ; Rutter et al., 2003) and a range of social descriptors which would enable the services pediatricians to screen the referrals to either accept for further assessment in the service or reject due to lack of evidence suggesting the presence of ASD.

Once accepted into the service, an ASD assessment included additional questionnaires completed by parent/caregiver and school, an appointment gathering an in-depth developmental history, and a BOSA. Prior to COVID-19, an ADOS-2 was part of the assessment. Children and their care givers completed the BOSA together whilst two ADOS-2 trained clinicians (community paediatricians, clinical psychologists, or speech and language therapists) observed either at a distance or through a one-way mirror. Clinicians chose the appropriate BOSA module for the assessment depending on the child’s age and language abilities. The outcomes of assessments were decided by the observing clinicians following the BOSA. Outcomes included diagnostic decisions (i.e., diagnosed, not diagnosed, tentative diagnosis and unable to conclude) and any subsequent decisions (i.e. additional information needed or onward referrals made).

As local COVID-19 infection control guidance was relaxed, clinicians were no longer required to socially distance from children attending clinic as long as face masks were worn. This raied the query over whether the BOSA was perceived as more helpful than an ADOS-2 wearing a face covering. In this instance neither instrument would be considered a validated measure.

### Participants and Procedure

#### Clinicians

Twelve clinicians working within the community paediatrics team took part in an online questionnaire about their opinions on the BOSA (see “Appendix A”). All clinicians were given a brief explanation of the project and consented to participate prior to completing the online questionnaire. The questionnaire comprised of three open-ended qualitative questions and eight quantitative questions which aimed to capture clinicians’ opinions on how helpful the BOSA is for making diagnostic decisions, and comparisons between the ADOS-2 and the BOSA. Two clinicians did not complete the qualitative section of the questionnaire. All clinicians were ADOS-2 trained (see Table 1), completed the BOSA training virtually, and had facilitated at least one BOSA prior to completing the questionnaire (see Table 2). The questionnaire was created via the online platform Qualtrics and sent to clinicians via email.

**Table 1:** Descriptive statistics representing how many years clinicians have been ADOS trained

How many years have you been ADOS-2 trained?	N (%)
0–2 years	4 (33)
2–4 years	2 (17)
4–6 years	1 (8)
6 + years	5 (42)

**Table 2:** Descriptive statistics representing how many BOSA clinicians had administrated at the time of completing the questionnaire

How many BOSA have you administrated?	N (%)
1–5	5 (42)
6–10	3 (25)
11–15	1 (8)
16–20	1 (8)
20 +	2 (17)

It is important to note the context in which this data was gathered and highlight that clinicians were far more familiar with the ADOS-2 compared to the BOSA. Many of the clinicians have four or more years of experience administrating the ADOS-2 (see Table 1), and yet only four clinicians had administrated the BOSA more than 10 times at the time of gathering the data (see Table 2). Furthermore, some may have more or less familiarity with the individual BOSA modules, creating further unfamiliarity with the BOSA as an assessment tool. The descriptive statistics regarding the four different module assessments completed by the clinicians can be found in Table 3.

**Table 3:** Number of children assessed in the four separate BOSA modules

Module	Total (N)	Mean age
BOSA-MV	3	3.70
BOSA-PSYF	19	5.16
BOSA-F1	63	8.08
BOSA-F2	6	8.5
BOSA-PSYF / F1 mix	1	6

### Analysis

#### Quantitative Data Analysis

Descriptive statistics were calculated using the quantitative data from the questionnaire with the aim of using these results to expand the findings of the thematic analysis.

#### Qualitative Data Analysis

The open-ended questions were analysed by the main researcher and second researcher using a thematic analysis approach, closely following the six-step approach of Braun and Clarke (2006). The researchers individually read the qualitative answers from participants multiple times before labelling individual data extracts and sorting these into codes. Rather than starting with preconceived notions of what the codes should be, an inductive approach to coding was chosen to allow the narrative to emerge from the qualitative data itself. The researchers discussed their individual coding and collaborated to create a codebook. This allowed the data to be read and re read, double checking the codes against the codebook, thus ensuring consistency and validity.

After this, codes with similar meanings were grouped together to form initial subthemes. Subthemes with similar information were then linked together which allowed the main themes to be developed. Once a consensus of themes was reached by the researchers, the answers that the clinicians gave were re-read to confirm the relevance of the themes and that they represented the original qualitative data.

### Ethics

Ethical approval was received from the Faculty of Medicine and Health Sciences Research Ethics Committee at the University of East Anglia (ethics reference code: 2021/22-020). Consent to complete the project within the local NHS service was granted by the trust’s Clinical Audit & Effectiveness Team.

## Results

### Quantitative Data

When asked to compare the BOSA to a standard ADOS-2, it was clear that clinicians found the BOSA less helpful for diagnostic decision making (see Table 4). No clinicians believed the BOSA is more helpful than the ADOS-2. It is important to highlight that this the standard ADOS-2 is a validated measure to use for ASD assessment, whereas the BOSA is not. It is therefore unsurprising that clinicians showed a clear preference for the ADOS-2.

**Table 4:** Clinicians opinions regarding the helpfulness of the BOSA for diagnostic decision making

Question	Much less helpful	Somewhat less helpful	Equally helpful	Somewhat more helpful	Much more helpful
Clinicians’ opinions on the helpfulness of a BOSA compared to a standard ADOS-2	N = 5 (42%)	N = 6 (50%)	N = 1 (8%)	N = 0 (0%)	N = 0 (0%)
Clinicians’ opinions on the helpfulness of a BOSA for diagnostic decision making compared to an ADOS-2 wearing a face mask	N = 5 (42%)	N = 3 (25%)	N = 1 (8%)	N = 3 (25%)	N = 0 (0%)

Most clinicians believed that completing an ADOS-2 with face masks was also more helpful for diagnostic decision making compared to a BOSA (see Table 4). However, 25% of clinicians believed a BOSA would be more helpful for diagnostic decision making compared to an ADOS-2 with face masks. In this instance, neither assessment method is validated. However, these results may still be expected, as the clinicians are highly familiar with the ADOS-2. Half of the participants have been using the ADSOS-2 for four or more years, whereas the BOSA has been used within the service for less than a year, with the majority of clinicians having only used the BOSA less than 10 times. Furthermore, some of the BOSA modules were scarcely administrated, again showing the lack of familiarity clinicians had with the BOSA.

However, interestingly clinicians did still show a preference to continue to administrate the BOSA in some instances. When asked if they thought the BOSA would be helpful to use even after COVID-19 restrictions were removed, 60% of clinicians answered yes, with the remaining 40% being split equally between no and unsure.

Clinicians’ confidence in diagnostic decisions after a BOSA were mixed (see Table 5), but the majority stated only sometimes their confidence in the diagnostic decision increased after a BOSA.

**Table 5:** Clinician’s opinions on whether the BOSA increased confidence in diagnostic decisions and the frequency that clinicians’ diagnostic decisions have changed after a BOSA

Question	Never	Rarely	Sometimes	Often	Always
Doing a BOSA increases my confidence in the decision to diagnose/not diagnose ASD	N = 1 (8%)	N = 2 (17%)	N = 6 (50%)	N = 3 (25%)	N = 0 (0%)
Doing a BOSA changed clinicians’ opinions around whether criteria for an ASD diagnosis has been met or not	N = 2 (17%)	N = 3 (25%)	N = 6 (50%)	N = 1 (8%)	N = 0 (0%)

Similar mixed responses were found when asking clinicians if doing the BOSA changed their opinions around their diagnostic decisions prior to the BOSA (see Table 5).

Five Clinicians disagreed that the BOSA was more time efficient compared to doing an ADOS-2. However, there were four clinicians who agreed that the BOSA can be more time efficient (see Table 6). This disparity in opinions was also captured in the qualitative data from the questionnaire.

**Table 6:** Clinicians’ opinions on the time efficiency of a BOSA compared to an ADOS-2

Clinicians’ opinions on the ASD assessment process being more time efficient using a BOSA compared to the ADOS-2	N (%)
Disagree	5 (42)
Somewhat disagree	0 (0)
No difference	3 (25)
Somewhat agree	2 (17)
Agree	2 (17)

### Thematic Analysis

The following themes were found and are presented in this section with supporting illustrative quotes from the clinicians (C). The themes are summarised in Table 7.

**Table 7:** Themes and sub-themes developed following thematic analysis

Higher order themes	Sub-themes
Administration of the BOSA	Parental administration issues Alternative administration by staff
Usefulness of the BOSA	Parent–child interaction observations Specific cases Time efficiency Materials, content and information gathering Using face masks

### Administration of the BOSA

Parental Administration Issues: Clinicians’ comments about parents and carers administrating the BOSA indicate that at times it can be difficult to get an accurate representation of the child’s communication and social interaction skills. In fact, seven of the 10 clinicians expressed parent administration of the BOSA can be detrimental for gathering necessary information for diagnostic decision making. This appears to be due to parents either being overly skilled at scaffolding their child during the BOSA (meaning difficulties could be suspected but not directly observed) or parents struggling with their own communication (impacting on the interaction opportunities)*.*

The main problem with the BOSA is that evidence for communication difficulties was not captured even though suspected due to amazing scaffolding that parents provide with their children. On other occasions, the BOSA was not useful due the inability of the parent to respond to child’s cues leaving the impression of a child with difficulties (C7)

…sometimes a parent can scaffold so well, the child does not present as autistic in the room (C1)

…if the parent is not ‘tuned’ into the assessment we do not always get the same information than from an ADOS (C5)

The parent delivered BOSA is very dependent on the skill and preparedness of the parent in how useful it is (C3)

Additionally, clinicians mentioned parent–child relationships and parental mental health can also impact the BOSA administration.

The BOSA can be negatively impacted by a difficult relationship with the administrator (parent/carer) and any of parent’s own social communication difficulties or anxiety about the observation (C8)

…a child’s interaction with their parent can be quite shut down (especially in cases of developmental trauma/parental mental health difficulties), but then in talking with them afterwards, they have demonstrated much better social communication (C10)

Alternative Administration by Staff: Some clinicians suggested the BOSA could be administrated by a professional, rather than a parent due to the complexities that parent–child interactions can bring.

BOSA could be run with one member of staff potentially. (C4)

If we are doing BOSA, I think it is important for a clinician to have a short time interacting with the child directly as well (C10)

where a child observation is still an important part of assessment, possibly delivered by a professional (C3)

In fact, one clinician stated they have had to take over the administration of the BOSA due to parents having difficulties administrating.

…on many occasions I had to step in as the parent had not been able to generate a "conversation" in a natural way. (C3)

### Usefulness of the BOSA

Parent–child Interaction Observations: Although clinicians felt parental administration of the BOSA could be detrimental for capturing the child’s communication and interaction skills, six out of the 10 clinicians mentioned the BOSA creates useful opportunities to observe parent–child interactions.

…the parent delivered BOSA can give useful information about the parent child relationship not usually available via the professionally delivered ADOS (C3)

I really appreciated the observation of the parent child interaction (C5)

It can be helpful to observe parent/child interaction which you do not get from the ADOS (C8)

BOSA good for understanding the parent-child relationship. (C10)

BOSA helpfully gives parental/ cater interaction and scaffolding (C4)

When clinicians were asked if they believed it would still be helpful to use the BOSA even if all COVID-19 restrictions were removed, six clinicians agreed that it would be helpful, of which four specifically stated that they would find the BOSA useful for observing parent–child interactions.

… if there is a particular reason to want an observation of parent/child interaction as it [the BOSA] allows a structured way to do this. (C8)

To observe parent child observation but I might do a combination of ADOS and BOSA to get a comprehensive picture of the child’s difficulties. (C5)

To observe the parent–child interaction, and how different the child’s communication is in that context to other contexts (C10)

BOSA gives very useful additional information about the dynamic between the child and the parent/carer that we otherwise wouldn’t see eg how parent interacts, parental ASD traits, if the parent scaffolds their child etc. (C1)

Specific Cases: Many clinicians stated the BOSA can be useful and appropriate tool in certain cases but not others.

…in some cases it [the BOSA] seems to be sufficient on its own provided you have a really good developmental history and school report. Depends on the child. (C1)

I think the BOSA is most helpful in quite straightforward cases and in the more subtle or complex I don’t think it gives good enough evidence to make a decision. (C3)

The BOSA seemed helpful for confirmation where the decision is reasonably clear from history/referral etc, but did not seem as helpful for making a decision for the children where the outcome is less clear. (C8)

BOSA was useful on a few occasions for children who are selectively mute (particularly if a one way mirror can be used). (C8)

Two clinicians mentioned the BOSA could be useful to continue to be used for specific cases even after COVID-19 restrictions are removed.

Straight forward cases where a child observation is still an important part of assessment (C3)

Not routinely but in particular circumstances e.g. as an option for children with selective mutism or if there is a particular reason to want an observation of parent/child interaction (C8)

Time Efficiency: Clinicians’ opinions varied when it came to the time efficiency of the BOSA. Although two clinicians mentioned the BOSA is a shorter and more time effective compared to the ADOS-2, this view was not shared by others. Some clinicians felt the lack of useful information gathered during the BOSA caused additional time to be spent getting information from other sources to make a diagnostic decision, thus reducing the efficiency of the BOSA.

BOSA is very brief and is much more time effective (C1)

Allow efficiency in clinic, allow us to see more patients (C4)

I don’t think the BOSA is much quicker than the ADOS when it comes to scoring and generating a report. (C3)

…this can lead to more time either chasing for further information or later bringing in for an ADOS. (C8)

Other clinicians expressed that the BOSA is too short for some children to feel comfortable or too short to spot repetitive behaviours, which perhaps would be observed naturally over a longer time period.

If BOSA actually administered in timeframe suggested, it is very short - a snapshot of interaction. but for some higher functioning kids with ASD it takes some time for the subtle difficulties to become more apparent, eg girl masking and the repetitive interests only appear over time in conversation (C10)

Not long enough for the child to warm up for anxious/ nervous children (C9)

Materials, Content and Information Gathering: Four clinicians specifically mentioned their preference for the BOSA materials compared to those in the ADOS.

I really like the BOSA toys, so much more engaging than awful out of date ADOS toys and games. (C1)

I think that the BOSA toys are more fun and often more age appropriate than the ADOS ones. (C5)

Better toys and games (C9)

I think using the BOSA materials for some of the ADOS tasks would be useful. (C10)

However, some clinicians believe the BOSA does not create enough opportunities for the child to demonstrate their level of understanding emotions and relationships or their ability to initiate social interaction.

I found the BOSA had few elements that allowed you to consider imagination and this was limiting compared to the ADOS (C3)

Bosa misses the more comprehensive understanding of emotions and relationships given by ADOS (C4)

It [the BOSA] is fun and more relaxing than the ADOS, but does not give opportunity to see how a child initiates interactions or seeks information. (C7)

Using Face Masks: Some clinicians brought up their opinions on the BOSA compared to conducting ADOS-2 using face masks. Of the four clinicians that mentioned using face masks, three were in favour of using the ADOS-2 with face masks over the BOSA. Although the ADOS-2 is not valid when using face masks, it is understandable that clinicians would prefer the more familiar assessment tools over the brand new BOSA, that is too not yet a validated tool.

Kids are so used to masks that an ADOS wearing a mask is good. (C6)

in my opinion the ADOS (wearing a mask) seems to give more information than the equivalent BOSA module (C8)

Of the children we have brought back in for an ADOS (with face mask) following BOSA almost all seem to be diagnosed with ASD…this highlights that the use of BOSA during the pandemic has led to delayed diagnosis for some children. (C8)

I do not think wearing a face mask impacts very much on the information we can get from an ADOS (which I feel is more than a BOSA) and I feel more confident making diagnostic decisions based on ADOS observation. (C10)

ADOS in face mask is not valid, I am not sure why we are doing these but my colleagues feel it is superior still to the BOSA. (C1)

## Discussion

In response to COVID-19 and mandatory regulations, the BOSA was rapidly developed to enable ASD assessments to continue safely. Due to some COVID-19 policies still being implemented within healthcare settings, the BOSA continues to be used by clinicians. Although the BOSA has been developed based on standardised activities from two well-validated assessments (the BOSCC and ADOS-2), the BOSA has not been validated itself (and at present no empirically derived cut-offs are available), thus caution should be taken when using the BOSA as a diagnostic tool (Lord et al., 2020; Rynkiewicz et al., 2020).

This project aimed to evaluate clinicians’ perceptions of how helpful the BOSA is for ASD diagnostic decision making in a community paediatrics team and clinicians wider opinions on the strengths and limitations of the BOSA. Two major themes developed from data: Administration of the BOSA and the usefulness of the BOSA.

Firstly, it is important to highlight that the clinicians recruited for this study were very familiar with the ADOS-2 (half of clinicians had four or more years of experience using the ADOS-2), and were only recently asked to learn, use, and evaluate the BOSA. It is plausible that the opinions in favour of the ADOS-2 are biased due to their knowledge and expertise of using the tool. However, despite this it is worth noting that clinicians had positive reactions to aspects of the BOSA, and these findings should not be undervalued. The results are discussed to highlight useful aspects of the BOSA and what may be missing from other ASD assessment tools such as the ADOS-2. Overall, the findings show mixed attitudes towards using the BOSA. One thing that is clear, is 92% of clinicians believe a standard ADOS-2 (without face masks) is more helpful for diagnostic decision making compared to the BOSA. This was an unsurprising result due to the ADOS-2 being a validated and reliable assessment tool which all clinicians had high familiarity with. A mixed opinion was found on the usefulness of an ADOS-2 using face masks compared to a BOSA, however the three clinicians that found the BOSA to be more helpful for diagnostic decisions compared to an ADOS-2 using face masks were in the minority. It is worth noting that an ADOS-2 with face masks is not standardised nor validated, and therefore cannot be scored accurately. Although the BOSA was created to fill the gap left by not being able to carry out a valid ADOS-2, it too is not a standardised, validated ASD assessment tool. In fact, Lord et al., (2020) encourage clinicians to rely heavily on a thorough developmental history, medical background, and parent report of symptoms due to the limitations and potential inaccuracy of the BOSA. Caution must be taken when using the BOSA to inform diagnostic decisions. Other telehealth ASD assessments which as validated may need to be considered such as the NODA or CARS-2.

When discussing the time efficiency of the BOSA, clinician opinions were mixed. The clinicians in this study highlighted that although the BOSA takes a short time to administrate, the inadequacy of the information gathered causes a greater demand on resources after the BOSA as clinicians are forced to acquire further information from other sources. This causes a delay for the children in receiving their diagnostic decision, but also increases the demand on resources in an already stretched service (NHS England, 2019). However, clinicians did acknowledge that for more straightforward cases, i.e., when a clear developmental history has been taken and ASD appears to be presenting from this, the BOSA can be more time efficient than the ADOS-2 and is helpful for confirming a diagnostic decision. If there are identifiable straightforward referrals that come into the service, completing a BOSA could speed up the diagnostic process, benefiting service resources and service users.

One of the main criticisms of the BOSA is that the brevity of the assessment reduces opportunities to observe certain behaviours and thus the likelihood for false negatives in diagnostic outcomes may be increased (Lord et al., 2020). Of course, a false negative ASD outcome can have a detrimental impact on children. If falsely given no diagnosis, children will not receive the adequate clinical and education support they may require and place responsibility for this entirely on parents (Charman & Gotham, 2013). Moreover, these missed diagnoses may cause individuals to seek help elsewhere for their difficulties, believing they may be due to anxiety or depression and thus further increase demands on mental health services (Aggarwal & Angus, 2015). Services must take this into consideration when deciding whether to continue to use the BOSA.

Furthermore, the validity and usefulness of the BOSA for clinicians appeared to be determined by the level of parental administration. For various reasons, the parent administration of the BOSA was highlighted by many clinicians as a barrier to gathering enough information for diagnostic decisions. So much so that one clinician mentioned that at times they had to actively get involved in the BOSA due to parents struggling to administrate effectively. Inability for parents to administrate the BOSA to a high enough standard ultimately invalidates the BOSA, making it harder for clinicians to assess the child’s social skills and behaviour, and thus further assessments may need to be completed to confirm diagnostic decisions. This then delays the children and their family from receiving an outcome. The longer it takes children and their family to receive a diagnostic decision the more parental stress increases, overall dissatisfaction of the diagnostic process increases and the longer it takes for children and families to receive appropriate support (Crane et al., 2016; Howlin & Moore, 1997; Mori et al., 2009). Delays in diagnostic outcomes also have a detrimental impact on the service. With one assessment taking on average 15 h of professionals’ time and costing £931 (Male et al., 2020), delays in outcomes will only take up more professionals’ time and ultimately cost the healthcare system more. This again is something to consider when services discuss the future of the BOSA or the potential use of alternative telehealth methods. Other virtual assessment tools such as the CARS-2 and TELE-ASD-PEDS are, like the BOSA, short to administrate, however the time efficiency overall when compared to the ADOS is unknown.

Some clinicians suggested the BOSA could be administrated by clinicians to address the barriers that parental administration can cause. This may increase the quality of information gathered and thus enhance the usefulness of the BOSA for diagnostic decision making. This may be an option if social distancing is no longer necessary, but some COVID-19 restrictions still apply (e.g., face masks), but if all restrictions have been lifted the standardised ADOS-2 can continue to be used.

Nevertheless, clinicians positively highlighted the opportunities that the BOSA creates to observe parent–child interaction. These observations allowed clinicians to gain insight into a wide range of parent behaviours (i.e., from high scaffolding to difficulties interacting) that could perhaps inform future support or interventions. For example, educating parents on how to adapt scaffolding in order to build their child’s social skills, or perhaps focusing on the parents’ own communication and interaction skills. Furthermore, observing parent–child interaction may be beneficial for clinicians to understand what a child finds most helpful and responds to best, to consider how this could be transferred into other settings, such as school. As well as the BOSA, other telehealth ASD assessments such as the NODA, CARS, TELE-ASD-PEDS and A-VABO also allow parent–child interactions to be observed if captured in the video content created by the family or during the videoconference. However, parent–child interaction is not something which the ADOS-2 accommodates, thus this useful information can be missed when using the assessment.

Clinicians also shared their preference for the BOSA toys and games compared to the ones used in the ADOS-2. However, some clinicians did mention the BOSA materials do not create opportunities to gather information about the children’s communication skills and understanding of social concepts, and so evidence for diagnostic decisions can be missed. This links to the potential risk of false negatives when using the BOSA (Lord et al., 2020).

Many clinicians thought the BOSA could be advantageous in specific cases, for example, children who are selectively mute. Over 60% of children diagnosed with selective mutism also have an ASD diagnosis (Cengher et al., 2021). In these cases, a parent administrated BOSA may allow clinicians to observe the child interact and communicate more than they would with an unfamiliar clinician during an ADOS-2.

The researchers acknowledge that this study does not come without its limitations. Firstly, the use of an online survey, as opposed to face to face or virtual interviews. Although the online survey was a more feasible method due to time restraints, it is plausible that interviewing participants would have collected more data and thus strengthened the findings of this study. Additionally, recruiting more clinicians may have improved the quality of this study. It must be noted that the conclusions of this study are based on a small number of clinicians’ opinions from a single service, and thus it is advised that future research explores this further.

Another limitation of the study is the use of two questions to ask clinicians to directly compare the usefulness of the ADOS-2 and the BOSA. In hindsight, the clinicians’ high familiarity with the ADOS-2 biases opinions when comparing it with a tool they have had minimal experience using. Furthermore, the BOSA is not yet a validated or standardised tool, thus it is inevitable that the ADOS-2 would be preferred by clinicians. Nevertheless, the finding that despite this some clinicians highlighted relative strengths of the BOSA compared to the ADOS-2 is a valuable finding.

Finally, this study did not complete any respondent validation. Again, this was not feasible due to time restraints of both researchers and clinicians. Future research should consider replicating this study in a larger service or across multiple ASD assessment services to explore clinician opinions on the BOSA further.

## Conclusions

Overall, the findings highlight some useful and beneficial aspects of using the BOSA. Clinicians expressed the usefulness of observing parent–child interactions during the BOSA, something that they are unable to observe when using the ADOS-2. Clinicians also noted that the BOSA can be more beneficial for certain cases such as selective mutism due to parental administration, rather than assessments which must be administered by a trained clinician.

The clinicians in this study showed a clear preference for the familiar ADOS-2 over the BOSA, even for using face masks during an ADOS-2. Both the BOSA and ADOS-2 with face masks must be used cautiously for diagnostic decision making, and clinicians should rely more on additional information from developmental history and parent/school report of symptoms than they may have done prior to COVID-19.

Clinicians should be wary of the risks of false negatives due to the brevity of the BOSA if it continues to be used. Future use of the BOSA may be beneficial for both children and services in certain circumstances, although it is evident that the BOSA should be properly validated if services rely on it for diagnostic decisions in these cases.

This study contributes to a growing body of literature on the BOSA and alternative ASD assessments used during and after the COVID-19 pandemic.

## Appendix

### Appendix A Clinician Questionnaire

#### BOSA Questionnaire

1. How many years have you been ADOS trained?

- 0–2 years
- 2–4 years
- 4–6 year
- 6 + years

2. How many BOSA have you administrated?

- 1–5
- 6–10
- 11–15
- 16–20
- 20 + 

3. Doing a BOSA increases my confidence in the decision to diagnose/not diagnose ASD.

**Table:**

1	2	3	4	5
Never	Rarely	Sometimes	Often	Always

4. Doing a BOSA has changed my opinion around whether criteria for an ASD diagnosis has been met or not.

**Table:**

1	2	3	4	5
Never	Rarely	Sometimes	Often	Always

5. Compared to a standard ADOS, I believe a BOSA is usually:

**Table:**

1	2	3	4	5
Much less helpful for diagnostic decision making	Somewhat less helpful for diagnostic decision making	Equally helpful for diagnostic decision making	Somewhat more helpful for diagnostic decision making	Much more helpful for diagnostic decision making

6. Compared to an ADOS wearing a face mask, I believe a BOSA is usually:

**Table:**

1	2	3	4	5
Much less helpful for diagnostic decision making	Somewhat less helpful for diagnostic decision making	Equally helpful for diagnostic decision making	Somewhat more helpful for diagnostic decision making	Much more helpful for diagnostic decision making

7. The overall ASD assessment process is more time efficient using a BOSA compared to the ADOS.

**Table:**

1	2	3	4	5
Disagree	Somewhat disagree	No difference	Somewhat agree	Agree

8. Please tell us more about your opinions of the BOSA. It may be helpful to consider:

- When you are unable to make a diagnostic decision from a BOSA, what information you feel you are missing?
- Any opportunities the BOSA gives you to collect further information that you can’t get from other methods
- Any factors you feel impact on how helpful a BOSA is?

9. (a) If all COVID-19 restrictions were removed, are there times when you believe it would still be helpful to use the BOSA?

- Yes
- No
- Unsure

(b) If you clicked “yes”, when do you feel a BOSA could still be useful:

10. Do you have any other comments about the BOSA?
